# Neuroprogression happens

**DOI:** 10.1017/S0033291724002149

**Published:** 2024-10

**Authors:** Eduard Vieta

**Affiliations:** 1 Departament de Medicina, Facultat de Medicina i Ciències de la Salut, Universitat de Barcelona (UB), c. Casanova, 143, 08036 Barcelona, Spain; 2 Bipolar and Depressive Disorders Unit, Hospìtal Clinic de Barcelona, c. Villarroel, 170, 08036 Barcelona, Spain; 3 Institut d'Investigacions Biomèdiques August Pi i Sunyer (IDIBAPS), c. Villarroel, 170, 08036 Barcelona, Spain; 4 Institute of Neurosciences (UBNeuro), University of Barcelona, Catalonia, Spain; 5 Centro de Investigación Biomédica en Red de Salud Mental (CIBERSAM), Instituto de Salud Carlos III, Madrid, Spain

I read with interest the contribution by Samamé on neuroprogression in bipolar disorder (Samamé, in press) in response to a commentary on the same topic (Vieta, 2024), and appreciate the opportunity to further discuss the nuances around the origin and course of cognitive impairment in patients with bipolar disorder from a scientific perspective.

The neuroprogression model fits well in some patients, but not all. When addressing cognition and functioning in conditions such as bipolar disorder, heterogeneity is the rule. But stopping and, ideally, preventing neuroprogression is the foundation of early intervention strategies, one of the most successful paradigm-shifts in Psychiatry (Martini et al., 2024). In her commentary, Samamé (in press) dismisses neuroprogression based on a study by Martino et al. (2013) that suggested that it was cognitive impairment what drove recurrences rather than the other way around. Unfortunately, that study, as the majority that do not support neuroprogression, used a very small sample (*N* = 70) of medicated, stable patients, with no comorbidities, followed-up for an extremely short period of time (16 months). Underpowered short-term studies of patients who are on medication since enrolment are extremely unlikely to reflect the natural history of illness (De Prisco & Vieta, 2024; Ilzarbe & Vieta, 2023). Although longitudinal designs are preferred to understand illness trajectories, sometimes it is better to rely on large and representative, well documented cross-sectional studies than on small, short-term single site prospective observations (Vieta & De Prisco, 2024).

Neuroprogression is neither a fact, nor a theory, that can be proven or disproven. It has little to do with causality, because it is about the ‘how’, not about the ‘why’, and I agree that correlation is not proof of causality; for the same reason, lack of correlation does not prove or discard anything. Psychiatry is a ‘soft’ scientific discipline, with borders with many other aspects of life that do not benefit from ‘black or white’ approaches. More than ever, the practice of Psychiatry today needs to be framed under the umbrella of ‘personalized medicine’ (Vieta, 2015). This means that in patients with bipolar disorder who show clinically relevant cognitive and functional decline it becomes paramount to establish a personalized, benefit-risk based strategy to stop illness progression, and that patients at early stages of illness should have the opportunity to embark in early intervention programs aimed at preventing further episodes, somatic and psychiatric emerging comorbidities, and cognitive decline. Nihilism and assumptions that the harm is already there do not help much.

In summary, despite the eloquent arguments of Samamé, I still think that neuroprogression does happen in some patients with bipolar disorder, as endorsed by numerous studies and some of the most active scientists in this field (Yatham et al., 2024), who explicitly argue that ‘evidence matters’. The neuroprogression model is definitely useful to predict outcome in a subset of patients and to intervene early, and when this is not possible, to provide relief through evidence-based psychotherapies such as cognitive and functional remediation, as well as psychoeducation. And it brings hope to patients, because it does not imply irreversibility, but rather a target for novel pharmacological and psychosocial treatments that may come up sooner than later through scientific innovation. But there is obviously the right to be wrong.
